# Association between Preferred Language and Risk of Severe Acute Respiratory Syndrome Coronavirus 2 Infection in Children in the United States

**DOI:** 10.4269/ajtmh.21-0779

**Published:** 2021-09-01

**Authors:** William R. Otto, Robert W. Grundmeier, Diana Montoya-Williams, Wanjikũ F. M. Njoroge, Kate E. Wallis, Jeffrey S. Gerber, Katherine Yun

**Affiliations:** ^1^ Division of Infectious Diseases, Children’s Hospital of Philadelphia, Philadelphia, Pennsylvania;; ^2^ Division of General Pediatrics, Children’s Hospital of Philadelphia, Philadelphia, Pennsylvania;; ^3^ Division of Neonatology, Children’s Hospital of Philadelphia, Philadelphia, Pennsylvania;; ^4^ Department of Psychiatry, Perelman School of Medicine at the University of Pennsylvania, Philadelphia, Pennsylvania;; ^5^ PolicyLab, Children’s Hospital of Philadelphia, Philadelphia, Pennsylvania;; ^6^ Department of Pediatrics, Perelman School of Medicine at the University of Pennsylvania, Philadelphia, Pennsylvania

## Abstract

The severe acute respiratory syndrome coronavirus 2 (SARS-CoV-2) pandemic has had a disproportionate impact on Black, Hispanic, and other individuals of color, although data on the effect of a person’s language on SARS-CoV-2 infection are limited. Considering the barriers suffered by immigrants and non-English-speaking families, we tested whether children with a preferred language other than English was associated with SARS-CoV-2 infection. Children from families with a preferred language other than English had a higher predicted probability of SARS-CoV-2 test positivity (adjusted odds ratio, 3.76; 95% CI, 2.07–6.67) during the first wave of the pandemic. This discrepancy continued into the second wave (adjusted odds ratio, 1.64; 95% CI, 1.10–2.41), although the difference compared with families who prefer to speak English decreased over time. These findings suggest that children from non-English-speaking families are at increased risk of SARS-CoV-2 infection, and efforts to reverse systemic inequities causing this increased risk are needed.

## INTRODUCTION

The severe acute respiratory syndrome coronavirus 2 (SARS-CoV-2) pandemic has excessively impacted Black, indigenous, and people of color, and persons of lower socioeconomic status.^1^^,^^2^ Prior reports revealed that Hispanic/Latino and non-Hispanic Black children had higher rates of hospitalization with COVID-19,^3^ and that 78% of pediatric deaths associated with COVID-19 in the United States occurred in Black, indigenous, and people of color children.^4^ In a recent multicenter retrospective cohort study, children with non-White race/ethnicity had lower rates of testing despite being more likely to have positive test results.^5^ Immigrant communities—particularly those with large numbers of essential workers and multigenerational households—have higher rates of SARS-CoV-2 infection.^6^ However, the association between a family’s preferred language and risks of SARS-CoV-2 infection in children has not been well described. Language data are documented in the electronic health record for the purposes of interpreter need, which presents an opportunity to evaluate the role of preferred language in COVID positivity. Therefore, we examined SARS-CoV-2 positivity rates across a large pediatric primary care network considering race/ethnicity and preferred language.

## METHODS

The Children’s Hospital of Philadelphia Care Network includes six primary care practices within Philadelphia. All practices share a common electronic health record (EHR) (Epic Systems, Verona, WI). Data related to caregiver preferred language, age, race/ethnicity, insurance status, and SARS-CoV-2 polymerase chain reaction testing were extracted from the EHR for all care episodes (including telemedicine visits) for patients younger than 19 years old from March 1, 2020 through February 28, 2021. Episodes of care that only included preventive services were excluded. Data from March 1, 2019 through February 29, 2020, were extracted as a comparison. SARS-CoV-2 tests performed as part of preadmission testing, pre-procedure screening, and scheduled screening for other reasons (e.g., for school or travel purposes) were excluded. This study was deemed exempt by the Children’s Hospital of Philadelphia Institutional Review Board.

Summary statistics were performed to describe SARS-CoV-2 testing results in the patient cohort. Demographic factors associated with SARS-CoV-2 test positivity were evaluated using multivariable logistic regression both with and without splines to adjust for temporal trends during the pandemic (R version 3.6.0, R Core Team, Vienna, Austria).^7^

## RESULTS

During the study period, 64,382 children received care in the designated practices. The overall patient volume and patient demographics were similar between pandemic and comparison periods (Table 1). Most patients were Black, and the preferred language for many families was English. For families with a preferred language other than English, the language most commonly indicated was Spanish.

**Table 1:** Care Network use before and during the pandemic and severe acute respiratory syndrome coronavirus 2 positivity by race/ethnicity, language preference, and insurance type

Variable	Primary care patients		SARS-CoV-2 testing, March 2020–February 2021		
	March 2019–February 2020 (*n* = 66,541)	March 2020–February 2021 (*n* = 64,382)	Tested (*n* = 10,138)	Positive (*n* = 1,284)	Positive within group (%)
Age, y; median (IQR)	6 (2–12)	7 (2–12)	5 (1–12)	9 (3–14)	–
Race/ethnicity					
White, non-Latino, *n* (%)	13,571 (20.4)	13,169 (20.5)	2,833 (27.9)	294 (22.9)	10.4
Black, non-Latino, *n* (%)	38,578 (58.0)	37,026 (57.5)	5,028 (49.6)	687 (53.5)	13.7
Hispanic/Latino, *n* (%)	5,581 (8.4)	5,534 (8.6)	978 (9.6)	159 (12.4)	16.3
Asian, non-Latino, *n* (%)*	2,971 (4.5)	2,809 (4.4)	406 (4.0)	59 (4.6)	14.5
Multiracial, non-Latino, *n* (%)	1,622 (2.4)	1,654 (2.6)	306 (3.0)	27 (2.1)	8.8
Other or unknown, *n* (%)†	4,218 (6.3)	4,190 (6.5)	587 (5.8)	58 (4.5)	9.9
Preferred language					
English, *n* (%)	61,292 (92.1)	59,199 (91.9)	9,542 (94.1)	1,171 (91.2)	12.3
Spanish, *n* (%)	1,604 (2.4)	1,560 (2.4)	227 (2.2)	51 (4.0)	22.5
Other, *n* (%)	1,318 (2.0)	1,320 (2.1)	185 (1.8)	31 (2.4)	16.8
Unknown, *n* (%)	2,327 (3.5)	2,303 (3.6)	184 (1.8)	31 (2.4)	16.8
Insurance					
Commercial/private, *n* (%)	28,624 (43.0)	27,137 (42.1)	4,737 (46.7)	539 (42.0)	11.4
Medicaid, *n* (%)	37,589 (56.5)	36,830 (57.2)	5,386 (53.1)	743 (57.9)	13.8
Unknown, *n* (%)	328 (0.5)	415 (0.6)	15 (0.1)	2 (0.2)	13.3

IQR = interquartile range; SARS-CoV-2 = severe acute respiratory syndrome coronavirus 2.

* Includes Pacific-Islanders.

† Includes Native American or Alaskan Native.

**Figure 1.**
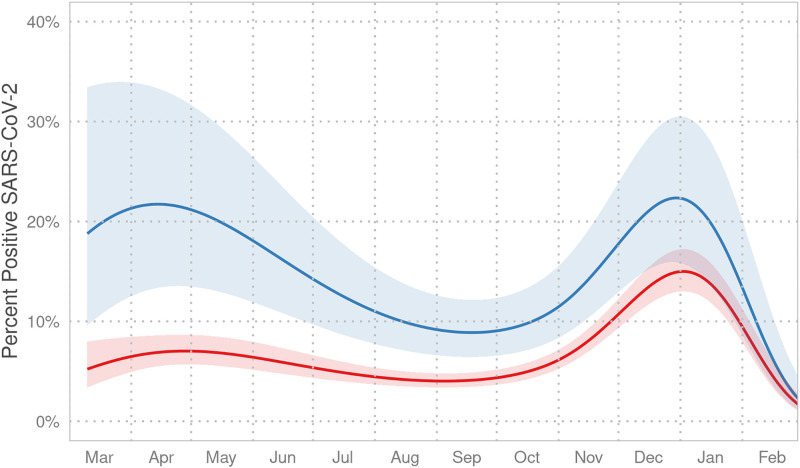
Spline-smoothed percent positivity over time from March 2020 to February 2021 for children in English-speaking families (red) vs. families who preferred a language other than English (blue). The shaded region represents the 95% confidence interval. The rates of positivity converged as incidence of COVID-19 decreased rapidly for all children in our region in February 2021. SARS-CoV-2 = severe acute respiratory syndrome coronavirus 2. This figure appears in color at www.ajtmh.org.

A total of 10,138 patients were tested for SARS-CoV-2, with 1,284 (12.7%) being positive. Test positivity was higher for Spanish-speaking families than for English-speaking families (22.5% versus 12.3%). In multivariable models adjusted for age and race/ethnicity, the predicted probability of test positivity was significantly greater for children with a preferred language other than English during periods of peak disease activity in our region (Figure 1). At the peak of the first wave of the pandemic, the adjusted odd ratio for test positivity was 3.76 (95% CI, 2.07–6.67) for families with a preferred language other than English compared with English-speaking families. The discrepancy had lessened by the peak of the second wave in December 2020 (adjusted odd ratio, 1.64; 95% CI, 1.10–2.41). The difference between groups decreased significantly in the time between the first and second waves of the pandemic (*P* = 0.03).

## DISCUSSION

In the urban primary care practices of a large pediatric health-care network in Philadelphia, children from families with a preferred language other than English were more likely to test positive for SARS-CoV-2 than children from families with English as a preferred language. This finding remained consistent throughout both local “waves” of the pandemic, although the difference between groups decreased over time.

These results are consistent with reported findings in adult patients. In a descriptive analysis of patients tested in the University of Washington medicine system early during the pandemic, the proportion of positive SARS-CoV-2 tests was 4.6-fold higher among patients with a preferred language other than English than English speakers.^8^ Similar findings were reported in patients of a large not-for-profit health-care system in the western United States, as having a primary language other than English was associated with a 2-fold increase in SARS-CoV-2 infections.^9^ Using data from the Massachusetts Department of Public Health, Figueroa et al.^10^ found that the factor most associated with elevated community risk of SARS-CoV-2 infection was the proportion of foreign-born, non-U.S. citizens. Foreign-born individuals also had elevated proportionate mortality rates compared with individuals born in the United States, in an examination of death files in California.^11^ Similar findings have been reported in other countries, including the United Kingdom and Canada.^12^^,^^13^ Together with our results, these studies highlight the disproportionate burden of the SARS-CoV-2 pandemic for immigrant families and families who prefer a language other than English, and support continued calls for large-scale interventions to reverse systemic inequities for those families.

Many factors increase risk of SARS-CoV-2 infection and poor outcomes of infection in non-English-speaking families. Working adults in immigrant families may be overrepresented in essential jobs, face difficulties advocating for occupational health protections, and have increased reliance on public transportation.^14^ Immigrant families frequently live in large, multigenerational households, increasing risk of transmission to vulnerable populations.^14^^,^^15^ Lack of access to preventative services increases the risk of developing chronic conditions associated with poor outcomes from COVID-19.^6^^,^^14^

Immigrants also lack adequate access to SARS-CoV-2 testing. Language barriers may prevent identification of appropriate testing sites.^16^ Other factors, such as distance to testing sites, need for an appointment, or perceptions that insurance or citizenship is needed, may also complicate an individual’s ability to get tested.^15^ Depending on immigration status, immigrants may avoid seeking medical care for fear of being deported.^10^^,^^17^ Immigrants may be excluded from unemployment or insurance benefits, and the financial ramifications of a positive test may be significant for individuals working for an hourly wage.^10^^,^^15^ These factors may prevent prompt presentation for care. In the absence of a positive test, these individuals are less likely to quarantine, preventing control of the spread of COVID-19.^15^ Finally, lack of access to language-concordant public health information may allow misinformation to spread and increase risk of virus transmission.^17^

To address these issues, every effort should be made to conduct large-scale studies of the impact of language on the risk for SARS-CoV-2 infection.^17^ Language data are often not collected during testing, and language is not a demographic field required by the Centers for Disease Control and Prevention.^18^ Improving the collection and tracking of preferred language will continue to be critical for testing processes to optimize contact tracing efforts and provision of individual guidance. More refined language data will also assist with targeted efforts to vaccinate individuals who prefer a language other than English.

Collaboration with trusted community stakeholders is essential to develop and disseminate pandemic-related public health information in languages that reflect local immigrant demographics.^9^^,^^12^^,^^13^^,^^17^ This will combat misinformation, improve general knowledge of SARS-CoV-2 and the current pandemic, and raise awareness about ways to combat the pandemic. Efforts should be made to improve availability to testing for SARS-CoV-2, including locating testing sites in affected communities. Last, efforts should be made to ensure that immigrants and non-English-speaking persons are included in vaccination rollouts. This will require continued engagement with these communities to identify possible barriers to vaccination and to work to encourage vaccine uptake.^19^

There are several limitations to this study. We compared positive tests from patients within a single health system within the United States. Our cohort was composed primarily of Blacks, and those with a preferred language other than English spoke Spanish predominantly. These findings may not be generalizable to other populations or clinical settings in the United States or other countries. In addition, we only captured positive test results from a single health system, and testing results in this study may not reflect the true positivity rate in our community. Although there are known limitations in how language is recorded in the EHR,^20^ the higher SARS-CoV-2 positivity we observed among children whose preferred language was not English is consistent with greater infection rates reported in such communities.^6^^,^^8^ EHR data generally under-identify patients whose preferred language is other than English—meaning, limitations in how language is recorded in the EHR would bias our results toward the null.

In conclusion, this is the first report examining the impact of family-preferred language on SARS-CoV-2 infection in children. We identified disproportionately elevated SARS-CoV-2 test positivity in children from families with a preferred language other than English, which is consistent with reported findings in adults in the United States and other English-speaking nations. As we continue to track and address the disproportionate impact of the SARS-CoV-2 pandemic on historically underrepresented and marginalized communities across the globe, it will be important to track disparities experienced by communities who speak languages other than English. Including such communities in risk mitigation strategies is not only the equitable approach, but also one that will assist in overall efforts to curb the pandemic and prepare for future pandemics.
